# Student and educator perspectives on virtual institutional introductory pharmacy practice experience (IPPE)

**DOI:** 10.1186/s12909-021-02698-5

**Published:** 2021-05-04

**Authors:** Omar A. Almohammed, Lama H. Alotaibi, Shatha A. Ibn Malik

**Affiliations:** Department of Clinical Pharmacy, College of Pharmacy, King Saud University, P.O. Box 2457, Riyadh, 11451 Saudi Arabia

**Keywords:** Experiential education, Pharmacy education, Pharmacy practice, COVID-19, Virtual training, Online learning

## Abstract

**Background:**

The COVID-19 pandemic has required governments around the world to suspend face-to-face learning for school and university students. Colleges of pharmacy are faced with the challenge of training students in hospitals that are under considerable pressure at this time. The government of Saudi Arabia has moved all classes and training online to limit the spread of the virus. This study describes the experience of the Introductory and Advanced Pharmacy Practice Experience (IPPE and APPE) students and preceptors engaged in the virtual IPPE training.

**Methods:**

A cross-sectional study was conducted to describe and appraise the implemented virtual IPPE training from the experiences of IPPE and APPE students, and their preceptor. The IPPE students described their experiences in close-ended questionnaires, while APPE students in open-ended questionnaires, and the preceptor described the experiences in narrative. The study focused on highlighting the advantages, opportunities, challenges, and shortcomings of the virtual training.

**Results:**

Two preceptors and seven APPE students participated in the preparation and administration of the virtual training. The IPPE students’ experiences, based on 87 respondents, were mostly positive. Although IPPE students enjoyed the time flexibility that allowed the learning of new skills and reflection on previous experiences, 15% experienced difficulty finding quiet places with a reliable internet connection or had difficulty working on team-based activities. Moreover, some were anxious about the lack of adequate patient-care experience. On the other hand, the APPE students found the experience enriching as they gained experience and understanding of academic workflow, gained skills, and overcame the challenges they faced during this virtual training experience.

**Conclusions:**

Future training programs should be organized to overcome the challenges and to maximize the benefits of training experiences. Schools of pharmacy may benefit from the training materials constructed, prepared, and administered by APPE students to improve IPPE students’ learning experiences and outcomes.

**Supplementary Information:**

The online version contains supplementary material available at 10.1186/s12909-021-02698-5.

## Background

The high transmissibility of severe acute respiratory syndrome Coronavirus 2 (SARS-CoV-2) led to the spread of COVID-19, which the World Health Organization categorized as a pandemic in March 2020 [1]. As a result, almost all affected countries underwent complete lockdown to limit disease transmission. In most countries, this included the closure of the entire education sector [2]. The implementation of non-pharmaceutical interventions, such as strict social isolation, and distancing measures, such as social distancing, case isolation, home quarantine, restrictions on mass gatherings, and the closing of schools and universities, are considered the primary preventive strategy to limiting the spread of COVID-19 until vaccines become available [3]. In April 2020, UNESCO reported that approximately 190 countries had implemented national school closure measures, affecting approximately 1.6 billion learners worldwide [4].

During the influenza outbreak caused by H_1_N_1_ in 2009, instead of closing schools, the government of Taiwan implemented a unique partial school closure policy to mitigate the spread of the virus while minimizing the potential social impact of full-scale school closure. Studies show that this policy effectively reduced the virus reproduction rate, and limited social disturbance [5]. Electronic learning (e-learning) was previously used during the 2002–2004 SARS-1 outbreak, and a study from Hong Kong found that students gained better results from e-learning classes than from traditional classes [6]. Meanwhile, the results of a European study indicate that virtual reality helps student learns better and more, and is expected to be developed for both autonomous learning and for the academic training of health science students [7].

According to international organizations, such as the Accreditation Council for Pharmacy Education (ACPE), one of the cornerstones of pharmacy education and training is the Introductory Pharmacy Practice Experience (IPPE). In response to the COVID-19 pandemic, ACPE has provided recommendations for alternative approaches to training in order to maintain standards while granting flexibility to those university programs providing this educational experience. The recommendations focus on alternatives that eliminate the need for the physical presence of the student [8]. Although virtual training is only recommended as a contingency, electronic training (e-training) has long been a viable option with the potential to resolve challenges facing IPPE even before the COVID-19 pandemic, such as the limited number of qualified preceptors and training sites [9, 10]. During the pandemic, hospitals and preceptors have been on the frontline, fighting the spread of the disease and providing care to affected patients, resulting in fewer sites than usual available to pharmacy students [11]. The resulting delay in graduation could lead to graduates missing out on opportunities to join residency programs or gain employment [8]. Therefore, the use of available resources for e-learning and e-training is now considered one of the options that can be utilized to minimize the impact of COVID-19 on learners and institutions worldwide [12].

In Saudi Arabia, the Ministry of Education decided to suspend all face-to-face classes and training and use available distance learning tools for teaching and training [13]. Given the uncertainty surrounding the persistence of Covid-19 and the probability of a second wave, similar to the 1918 “Spanish” influenza pandemic [14], the College of Pharmacy at King Saud University (KSU) decided to move to virtual training using all resources at its disposal. The utilization of pharmacy APPE students as teaching assistants had previously been found to be effective, leading to increased course efficiency and maintaining support staff costs at a reasonable level [15]. Therefore, as part of their academic rotation, APPE students were involved in the preparation and administration of the virtual training experience, thus providing them with the valuable learning experience of performing all required academic teaching activities.

The current research was conducted to document the experience of virtual format institutional-IPPE from a number of perspectives. It was aiming to evaluate the experience of pharmacy students taking virtual institutional-IPPE, and the experiences of APPE students and preceptors in its preparation and administration. By assessing the experiences, benefits, and challenges of virtual IPPE training, the study can provide a new training model, so that other schools can learn from the KSU experience.

## Methods

### Study design and data collection

A cross-sectional study was conducted to describe and evaluate the implemented virtual IPPE training from the perspectives of IPPE students, APPE students (who participated in the preparation and administration of this training), and their preceptors. The experience of IPPE students was assessed using a close-ended web-based Google Forms questionnaire (IPPE Students’ Experience Questionnaire) administered immediately after completion of training and once students had received their final training grade. The experience of APPE students was evaluated through the qualitative analysis of an open-ended questionnaire administered by email at the end of their rotation (APPE Students’ Experience Questionnaire). The experience of these students’ preceptors was reported as a narrative explanation of the design, preparation, and assessment for the virtual IPPE training and their personal experience of the training. Participation in the study was voluntary, and IPPE and APPE students were sent frequent reminders encouraging them to complete the questionnaires. The study design and questionnaires were reviewed and approved by the Institutional Review Board at King Saud University Medical City (Ref. No. 20/0062/IRB).

### Participants

A total of 130 (77 male and 53 female) fourth- or fifth-year B. Pharm and Pharm. D students were eligible to enroll in the virtual IPPE training. The IPPE training program was designed, prepared, and administered by seven APPE students under the guidance and support of two IPPE preceptors. IPPE and APPE students were invited via e-mail and text message to participate in the study and complete a questionnaire. One IPPE preceptor (OAA) documented his experience and the other preceptor revised and approved the narration based on her experience; this section was added in order to ensure a complete description of the virtual training from all perspectives.

### Questionnaire

The IPPE Students’ Experience Questionnaire was designed to assess students’ experiences with the virtual training. It contained 48 items divided into two sections. Section one included eight items designed to capture demographics and the impact of the pandemic on the personal lives of the students, while section two included 40 items designed or adopted to assess the students’ experiences with the virtual IPPE training. Thirty-eight of the items were measured on a five-point Likert scale and two were ranking questions. The Likert scale questions measured perceived benefits and experiences with virtual training, the difficulties and challenges faced during virtual training, opportunities that arose during virtual training, and the shortcomings of virtual training. The final four items were adopted from the Brief Resilient Coping Scale (BRCS) to capture the students’ stress-coping tendencies [16]. Meanwhile, the ranking questions were used to gauge preferred learning and assessment styles.

The APPE Students’ Experience Questionnaire was designed to assess APPE students’ experiences of the virtual IPPE training. It consisted of 25 open-ended questions in which respondents could freely describe their experiences, including challenges faced, opportunities encountered, and skills and knowledge gained or developed during each stage of the IPPE training preparation and administration process. It also included questions about the IPPE training as a whole, gauging opinions about different educational styles and assessment methods, if they would participate in or recommend virtual training in the future, and the reasons for their answers. This section was designed with open-ended questions aiming to cover all aspects under investigation to allow the performance of an inductive content analysis that identify themes, while confirming that collected data were credible in terms of covering the APPE students’ distinctive experience. Due to the limited number of APPE students, efforts were made in the data collection step to ensure that all APPE students would participate to allow reaching a saturation level in the data analysis stage.

### Data analysis

Descriptive statistics, including mean with standard deviation and frequencies with percentages, were used to describe IPPE students’ characteristics and virtual training experiences. Data were coded and analyzed using SAS software, version 9.4 (SAS Institute Inc., Cary, NC). Qualitative data analyses were used to assess APPE students’ experiences of preparing and administering of virtual training. Responses were preserved in written form, and no transcription or software was required or used for analysis. The main aspects of investigation among the APPE students in the qualitative questionnaire were experiences, challenges faced, opportunities encountered, and their opinions of virtual training and its components. When performing the inductive content analysis and to ensure the credibility of the results, two researchers (LHA and SAI) have read each of the APPE students’ responses independently, extracted key words that were used as the unit of analysis to represent repeating ideas, and created a table to assign colors for each key word (e.g., “Time limitation” was assigned as red and covered any statement that indicate difficulty completing tasks on time, meeting deadline, or any other time relating difficulty). A consolidated document was created and shared between the researchers in which the items on the questionnaire and the corresponding answers for each item from all participants were organized in tables. Then, similar ideas were codded using color-coding technique while referring to the key table. After reviewing the consolidated color-coded file, general themes under each aspect were identified upon agreement between the two investigators (LHA and SAI) and validated by the third (OAA) to ensure the credibility or conformability of the findings. As color-coding was used to group similar ideas and those ideas represented broader themes, some answers were selected to be representative for each theme while ensuring the incorporation of quotation from multiple participants to support the authenticity of each statement. Besides that, few items had distinctive responses that were extremely specific and did not fit under common themes among the participants (e.g., *would you join or recommend online training courses in the future to others? Why?*); thus, the responses to such item were coded in accordance as either affirming or negating the statement in these items.

## Results

### The preceptor’s experience of the virtual IPPE training

#### The decision to offer virtual IPPE

Hospital training of pharmacy students has always been a challenge, but the COVID-19 pandemic transformed it from a challenge to a possible threat to schools, students, and training sites. A number of factors were assessed before the decision was made to move to virtual training, rather than continuing with or entirely postponing training. These factors were ranked and listed in Table 1 to assist future decision-making regarding pharmacy or medical field training in similar situations. Having reviewed these factors and assessed the appropriateness of available options based on previous and current experience, the college decided that it would be in the best interests of the students, training sites, and the college to:

1. offer the institutional-IPPE training in a virtual training format, and
2. postponing the community-IPPE training to next year.

**Table 1:** Factors assessed before the decision to move for virtual IPPE training

♦ **College-related factors**	
• Availability of sites, seats, and preceptors for the IPPE training.	
• Fear of placing students at risk or being responsible for the spread of infection to families/relatives.	
• Fear of being responsible for the spread of the virus in the community.	
• Uncertainty of the pandemic continuity and its impact on current and succeeding student training.	
♦ **Student-related factors**	
• Fear of being infected by a virus for which no curative treatment is available.	
• Fear of delaying their graduation.	
• Fear of compromising their training experience.	
♦ **Site- and preceptor-related factors**	
• Exceeding the capacities of training sites in the post-pandemic period.	
• Spreading infection among staff of training sites.	
• Limited utilization of resources for the procurement of personal protective equipment (PPE).	
• Limited availability of PPE in training sites.	
• Limited number of staff (i.e., preceptors) working on-site to satisfy social distancing parameters.	

While community-IPPE training was postponed, the curriculum and graduation of the students eligible to take that training were not affected, nor were the students in the succeeding cohort. This is mainly due to the availability of a greater number of sites and preceptors for community-IPPE, meaning that the college did not face the challenge of finding sites for student training. Therefore, these students had the opportunity to live the actual rather than the virtual experience.

#### Design and construction of the IPPE training

The institutional-IPPE training is normally offered as a full time (5 days/week, 40 h), four-week training program during the summer semester for students who have completed their fourth academic year. It is also offered as a part-time (2 days/week, 16 h) 12-week training program during the second semester of the fourth academic year, to overcome the issue of limited site and seat availability. The institutional-IPPE virtual training program was offered during the 2020 summer semester, utilizing the normal school learning management system (LMS) as the main portal for the presentation of all training materials and to document all student activities and assessments. It was designed to cover most of the objectives of the actual institutional-IPPE, while some of the objectives that require direct patient interactions (e.g., collecting patients’ past medical history) or actual presence (e.g., exhibiting good communication skills and demonstrating a high standard of professional behavior), which were not possible to perform or assess in virtual setting, were replaced by other objectives to complement the shortage in the virtual training. The objectives for the actual and virtual training were listed in Table 1A of the supplementary material.

The educational training materials were offered over a three-week period, and each week addressed objectives related to the main theme for the week, including the outpatient pharmacy, the inpatient pharmacy, and pharmacy management, in addition to training on the necessary soft skills of pharmacy practice. The virtual training was constructed to have the trainees:

1. attend pre-recorded lectures and/or videos and then complete corresponding individual or team-based online assessment activities;
2. attend and complete multiple mini online courses (1–5 h) offered by other national and international institutions/organizations; and
3. engage in clinical activities and solve cases designed on a pharmacy simulation system.

All lectures, videos, online courses, and simulation-based experience, or online assessment activities, were offered in an asynchronous style and the IPPE students were required to complete all activities within a four-week period. This was decided bearing in mind technical issues that might arise and to be considerate of trainees who might contract the virus and who would, therefore, require rest for a few days. The overall assessment of the IPPE training was graded as usual for the actual training, i.e., pass/fail grade, and students who completed at least 80% of the online courses, simulation experiences, and assessment activities (with a score over 70% in each activity) were considered to have met the minimum requirements to pass the IPPE training.

#### The role of pharmacy APPE students in virtual IPPE training

After specifying the objectives for each week of the virtual training program, APPE students were involved in a number of steps, from selecting or preparing training materials to assess student performance and preparing a final report to submit to the College’s Academic Affairs section. The APPE students were heavily involved in suggesting the types of activities that could be performed to achieve each objective and in preparing student assessment activities. Their suggested materials and activity plans were then reviewed and approved by the IPPE preceptors. Once approved, the APPE students prepared lectures or utilized available online resources to select appropriate videos of pre-existing lectures (e.g., lectures from international organizations available on YouTube). Pharmacists from the university-affiliated hospital were invited to prepare lectures or instructions to fulfill certain objectives for which APPE students lacked knowledge or experience. The process of preparing and administering the virtual training program and the roles for the APPE students in this process were presented in Additional file 1: Fig. 1A.

To facilitate communication between APPE students and specify the tasks and responsibilities of each, the seven APPE students were divided into three teams. Each team was responsible for the plan and content for their assigned week and for the administration of the IPPE training and assessment of activities on the LMS. All these tasks were completed while working under the direct supervision of the IPPE preceptor (OAA), who was the preceptor for their academic rotation.

#### The experience of working on virtual training with APPE students

The engagement of the APPE students in the training of their fellow students was a success for all involved parties. The APPE students learned about the nature of academic work, were enthusiastic about contributing to their fellow students’ training, and learned how to construct and administer a training program. Moreover, they learned, through the first-hand experience, about the challenges and opportunities of virtual learning and training. Without the inclusion of APPE students, the administration of the virtual training program would have been very challenging for the IPPE preceptors, and the quality of the training might have been compromised. However, by involving APPE students, the IPPE preceptors were able to set high goals, even though it was their first time to be involved in the development of a virtual IPPE training program. The preceptors believe that the IPPE training would not have been a success without the participation of the APPE students. Neither the college nor the university had to shoulder any additional costs for the administration of virtual training or delay the graduation of either IPPE or APPE students. Institutional-IPPE training was offered as planned and without delay. Without this, the college would have faced a big challenge in training the succeeding student cohort. The IPPE students completed their institutional-IPPE training without heavily compromising their experience. Therefore, neither the graduation of B. Pharm students nor the start of internship training for Pharm. D students were delayed.

### IPPE student experiences with the virtual training

Among the 130 students registered in the IPPE training, two students decided to drop the training when they found out that it would only be offered in a virtual format. Therefore, the end-of-training questionnaire was distributed to 128 students, of which 87 responded. The overall response rate was 67.9% (70.7% for males and 64.2% for females). The average age of IPPE students was 22 (±0.76) years, and 39.1% were female. Only 11.5% of participants needed to change their residence due to the pandemic, while 10.3% needed to change their residence to enable enrolment in the virtual IPPE training, and 13.8% lost part-time jobs due to the pandemic. The students’ tendencies to cope with stress ranged between 9 and 20, with a mean of 15.1 (±2.8). Participant characteristics are presented in Table 2.

**Table 2:** IPPE student demographic, education, and living condition characteristics (*n* = 87)

Characteristic	Frequency
Age, mean (SD)	22 (0.76)
Gender	
Male	53 (60.9)
Female	34 (39.1)
GPA, mean (SD)	4.46 (0.43)
Educational program	
B. Pharm	23 (26.4)
Pharm. D	64 (73.6)
Usual living condition during the academic year	
Usually living with parents and/or siblings	77 (88.5)
Usually living alone	6 (6.9)
Usually living with spouse	4 (4.6)
I had to change my residence because of the pandemic	10 (11.5)
I had to change my residence because of the virtual training	9 (10.3)
I have lost my part-time job due to the pandemic	12 (13.8)
Students’ tendency to cope with stress (from BRCS), mean (SD)	15.1 (2.8)

The values in the table are frequency (%), unless otherwise specified.

In response to questions about their experience of the virtual training program, two-thirds were happy that their training continued without delay and 48.3% indicated that the number of activities involved was enough to learn new skills, but only 39% were happy that their training would be virtual. Around three-quarters found the electronic LMS to be user-friendly and agreed that it was successfully used in virtual training. A similar proportion enjoyed participating in the implemented simulation activities and agreed that these activities provided them with experiences close to real-life training. More than 60% indicated that their overall experience was good and they were satisfied with what they had learned, the electronic resources were beneficial in their virtual training, the time allowed for completing the required activities was adequate, the training coordinators were readily available to answer questions and concerns, and the experience provided an environment to facilitate learning and enhance their clinical and written communication skills, but not their verbal communication skills. Around 70% were able to apply what they had previously learned and they believed that this experience will help them become better pharmacists in the future. The dispersion of the participants’ responses to the experience items is shown in Table 3.

**Table 3:** IPPE students’ experiences with the virtual IPPE training

Items on the questionnaire	Strongly disagree	Disagree	Neutral	Agree	Strongly Agree
I was excited when I learned that my training would not be delayed	4.6	6.9	21.8	20.7	46.0
I was happy when I learned that my training would be conducted virtually	14.9	18.4	27.6	19.5	19.5
The goals and objectives of the training were outlined and/or explained at the beginning of the training	1.1	2.3	23.0	34.5	39.1
The use of the electronic learning management system for training was successful and user-friendly	4.6	6.9	11.5	37.9	39.1
The use of simulation was enjoyable and provided me with close experience to real-life training	1.2	2.3	21.8	28.7	46.0
The electronic lectures and resources provided were beneficial in my training	4.6	5.7	27.6	41.4	20.7
The time allowed for completing the required activities was adequate	2.4	8.0	23.0	33.3	33.3
The quantity of activities that needed to be completed on a weekly basis was sufficient to learn new things	4.6	17.2	29.9	34.5	13.8
The online training coordinators were readily available to answer questions and concerns	2.3	4.6	24.1	36.8	32.2
This virtual training experience provided an environment that facilitated my learning	4.6	4.6	27.6	37.9	25.3
My verbal communication skills were enhanced by this training experience	9.2	12.6	35.6	32.2	10.4
My written communication skills were enhanced by this training experience	4.6	5.7	23.0	48.3	18.4
My clinical skills were enhanced by this training experience	5.7	5.7	26.4	46.0	16.1
I was able to apply previously learned materials during this training experience	2.3	4.6	23.0	46.0	24.1
My overall training experience was good and I am satisfied with what I have learned	2.3	8.0	21.8	40.2	27.6
I believe this experience will help me to be a better pharmacist	3.4	6.9	16.1	49.4	24.1
The use of online quizzes, completing online courses, participating in team-based activities, and simulation activities was a fair way to assess my learning experience	6.9	5.7	34.5	29.9	23.0

The values in the table are percentages of the participants in each category.

With regards to the difficulties IPPE students faced, approximately 15% reported that it was difficult to find a quiet place to work with a reliable internet connection. Only 11.4% reported difficulties communicating with their training coordinators, but 18.3% indicated that it was difficult to meet their classmates and work on team-based activities. Only 5% found receiving constructive mid-point and final evaluations on their performance difficult. The difficulties that IPPE students experienced during their virtual training are presented in Table 4.

**Table 4:** Difficulties that IPPE students experienced during their virtual training

Items on the questionnaire	Not difficult at all	Not difficult	Neutral	Difficult	Very difficult
Finding a place with a reliable internet connection	36.8	21.8	28.7	7.0	5.7
Finding a quiet place to attend a training session, or work on training activities	29.9	20.7	32.2	11.5	5.7
Communication with my instructors or preceptors	24.2	33.3	31.1	5.7	5.7
The ability to virtually meet my classmates to work on team-based activities	20.7	37.9	23.1	14.9	3.4
Access to online courses, quizzes, simulation activities, or presentations	32.2	37.9	19.5	9.3	1.1
Receiving constructive mid-point and final evaluation for my performance throughout the training	32.2	37.9	25.3	4.6	0.0
The use of multiple distance learning platforms in addition to outside sources, such as YouTube®, and websites for online courses	34.5	33.3	20.7	10.3	1.2

The values in the table are percentages of the participants in each category.

The IPPE students were also asked about the benefits and opportunities that the virtual training format offered. The majority was able to reflect on what they were learning and what they want to do in the future (65.5%), and had more time to learn new skills (55.2%). Nearly 65% believed their experience with distance training has improved and are encouraged to use distance learning and training in the future. Most (64.3%) liked the flexibility of virtual training and about 30% reported having time to volunteer or contribute to their communities during the training period. The opportunities or benefits of IPPE virtual training are summarized in Table 5.

**Table 5:** Benefits and opportunities that IPPE students experienced during their virtual training

Items on the questionnaire	Strongly disagree	Disagree	Neutral	Agree	Strongly Agree
I had more time to learn new skills	2.3	6.9	35.6	36.8	18.4
I liked the time flexibility when working on my training activities	1.1	9.2	25.3	28.7	35.6
I believe that my experience with distance learning and training has improved	1.1	8.0	25.3	37.9	27.6
I feel encouraged to use distance learning and training more often in the future	8.0	3.4	24.1	37.9	26.4
I was able to reflect on what I was learning and what I want to do in the future	3.4	4.6	26.4	43.7	21.8
I was able to perform patient care related activity in a way that will help me in the future	0.0	9.2	28.7	43.7	18.4
I had time to volunteer in projects or initiatives that helps the community	10.3	14.9	44.8	23.0	6.9

The values in the table are percentages of the participants in each category.

When asked about perceived shortcomings related to the virtual format of the IPPE training, only 25% indicated that they did not receive all of the training experience they had expected, and 16% were unable to complete the training tasks. Although over 60% of students believed they could now perform patient care-related activities in a way that will help them in the future, 43% were afraid that they lack enough direct patient care experience. The students’ perceived deficiencies in virtual training are presented in Table 6.

**Table 6:** Deficiencies in virtual training from the IPPE students’ perspective

Items on the questionnaire	Strongly disagree	Disagree	Neutral	Agree	Strongly Agree
I have not received all the training experience that I was expecting to receive	17.2	31.1	26.4	18.4	6.9
I am afraid that I may not have enough direct patient care experience	2.3	19.5	34.5	26.4	17.2
I was not able to learn or complete training tasks that I was working on	28.7	32.2	23.0	11.5	4.6

The values in the table are percentages of the participants in each category.

**Fig. 1:**
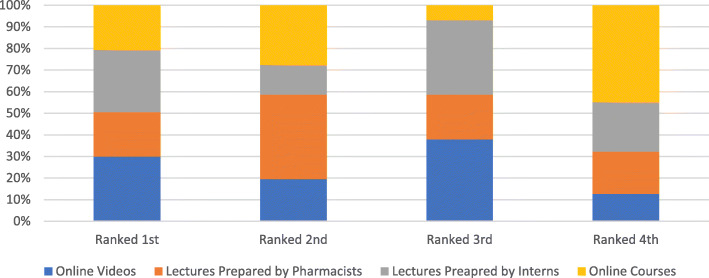
Ranking of learning method based on the IPPE students’ preference 1

**Fig. 2:**
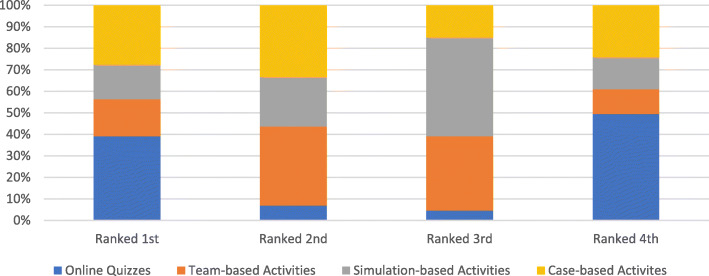
Ranking of assessment methods based on the IPPE students’ preference

With regard to learning and assessment methods, 30% of respondents ranked online videos and lectures prepared by APPE students as their preferred learning method. Lectures by pharmacists from the affiliated medical city were most likely to be ranked 2nd, while 45% ranked online courses as their least preferred method. Preferences for assessment methods varied significantly. Most notably, 40% ranked online quizzes as their preferred choice, while 50% ranked them as their least preferred choice. Case-based activities were ranked 1st by 25% and 2nd by 32%, team-based activities were ranked 2nd by the greatest number of respondents, and simulation-based activities were ranked 3rd by approximately 45% of students. Figures 1 and 2 represent students’ learning and assessment method preferences, respectively.

### APPE students virtual IPPE training experiences

APPE students described their overall experience while preparing the IPPE training plans and materials as enjoyable but with challenges for both themselves and their preceptor. Time constraints and access to online resources through the school system were serious challenges for most. Some APPE students did not have a clear understanding of the objectives of their extraordinary rotation and lacked guidance when searching for suitable training materials. Regardless of these challenges and difficulties, the APPE students managed to deliver good training materials and activities for their fellow students. To achieve this, they utilized their own recent experiences as students and used what they had learned from their own IPPE training, pharmacy practice lab, and other courses to construct materials and assessment activities that they would have liked for themselves.

Delivering virtual training provided many opportunities for APPE students in their academic rotation, most particularly the opportunity to understand the academic workflow. It also gave them the opportunity to provide students with suitable plans that will benefit them in the future. Moreover, the APPE students described their experiences of presenting the materials, constructing online assessments, and coordinating the virtual training as easy and entertaining. This was especially the case because of the availability of video tutorials explaining how to use the school LMS, which all APPE students agreed was user-friendly.

The APPE students reported that time management skills, computer skills, communication skills, teamwork, and teaching skills were the most beneficial when constructing and administering virtual training. They also believed that, in addition to these skills, they developed patience, creative thinking, and problem-solving skills. The APPE students found the pharmacy simulation program to be a great experience, and consider it a step forward, as practice is a valuable way to improve learning and training outcomes, and it helps students to visualize the medication dispensing process. Therefore, all APPE students said they would recommend that it be used in future training.

Although APPE students learned to give constructive feedback as a part of the student assessment process, but they struggled with giving grades. In the absence of a grading rubric, they found it difficult to ensure grading fairness. However, having model answers at their disposal helped them to provide proper assessment and appropriate feedback.

When discussing the expected preferred learning styles for IPPE students, many APPE students chose simulation and lectures; the former, because it is a new creative method that provides realistic scenarios and engages the students, and the latter because of its familiarity. APPE students had different views on the assessment of student performance. The majority believed that multiple-choice quizzes were the best assessment method to measure students’ understanding of the materials. However, others thought that simulation and team-based activities were the best, as they provide more realistic assessment.

Finally, there was great variation in APPE students’ responses to the question, “Would you join or recommend online training courses to others in the future?” Some were highly supportive, while others were completely against online training programs. Examples from the APPE students’ responses were provided in Table 2A of the supplementary materials.

## Discussion

This study was conducted to assess the experiences of IPPE and APPE students and to share what have been learned from this experience with other schools that might be facing similar challenges. The study sought to explore the advantages, opportunities, and challenges faced during the development and implementation of virtual training programs for pharmacy students. Hopefully, the findings of this study will prove useful for other health profession training programs. Overall, the majority of IPPE students reported a positive experience. They enjoyed the flexibility offered by virtual training, which gave them the time to learn new skills and to reflect on what they were learning, as well as what they might want to do in the future. Most of the IPPE students acknowledged that their virtual training enabled them to perform patient care-related activities in such a way that it will help them in their future practice. However, some were anxious that the virtual training had not provided them with enough direct patient care experience. Only one-fifth of students indicated that the number of activities was insufficient to learn new hospital-related materials and their verbal communication skills were not enhanced. Although was not very common (~ 15%), finding a quiet place with a reliable internet connection or working virtually on team-based activities were the most frequent difficulties cited by students. Online courses were the least preferred method of learning, and online videos and lectures prepared by APPE students were the methods favored by most students. While students had opposing views on online quizzes as a method of assessment, the majority preferred case-based and team-based activities over simulation-based activities. APPE students reported an overwhelmingly positive experience as they experienced and developed an insider understanding of academic workflow and had the opportunity to prepare good training materials for their fellow students. Moreover, they gained valuable skills during this new experience and overcame a number of challenges. However, some of these challenges could have been avoided if more time had been available for planning, constructing, and administering the IPPE training and if the school had ensured continuous access to resources.

Having a reliable internet connection is one of the most important resources in virtual training. A small proportion of students indicated that it was difficult for them to have reliable access to the virtual training. Access to online resources and virtual training was cited as a concern for educational institutions in a recent paper discussing experiential pharmacy education during the COVID-19 pandemic [17]. In that study, almost 13% of students had difficulty finding a reliable internet connection, while another Saudi study found that 21% of students across 19 different colleges faced the same issue [18]. However, one qualitative study exploring medical students’ perspectives of online learning suggests that students were able and motivated to fulfill course requirements in less time using educational technology. Similar to the current study, it also found that clinically-oriented fourth- and fifth-year students find online classes highly beneficial, and prefer recorded lectures to face-to-face lectures because the former offer flexibility for the students to return to them as needed and their time-saving benefits improve student performance [19]. That study also touched on the universal challenges facing online learning, such as communication and internet connectivity issues. However, overall, the majority of students in that study preferred online classes. Therefore, the use of virtual training can be useful, at the very least, as a supplement to actual training to enrich the student experience. However, it should not be considered a complete alternative for real-world experience and should allow students with poor access to reliable internet, to delay their training.

Most of the IPPE students recognized that one of the weaknesses of the virtual training program was inadequate direct patient care experience. Unfortunately, given the circumstances, the incorporation of actual or virtual direct patient care activities was not an option, due to the limited preparation time, a large number of trainees, and the lack of preceptors. Instead, simulated activities were designed and prepared by APPE students and preceptors to provide IPPE students with at least partial direct patient care experience. These activities were implemented to emulate medication validation and dispensing as well as providing patients with counselling points and recommending over the counter medications. Another option that could improve the IPPE students’ learning and experience would be the incorporation of telemedicine to provide students with direct patient care experiences to help them develop confidence in their clinical skills. This was implemented by Virginia Commonwealth University’s Richmond Health and Wellness Program and they concluded that telemedicine might be inadequate for training programs with large numbers of trainees [20]. In their experience, telephonic visits allowed only a few students to maintain effective communication with patients. Because student teams were reduced from four or five students to only two per visit, only 62 students could participate in telephonic visits over the summer, compared to the usual 100 students per semester [20]. Taking their experience into consideration, future virtual training programs with limited number of trainees or large number of preceptors can use telemedicine, in addition to other forms of virtual training, to provide students with more patient care experience.

In the virtual training program, the students did not have access to electronic medical records and the other benefits of traditional training, such as practicing professional verbal and non-verbal communication while providing care to patients or when interacting with other health care professionals. This was one of the challenges highlighted in the above-mentioned paper discussing experiential pharmacy education during the COVID-19 pandemic [17]. As a result of the lack of access to patient care, some students worried they might not have enough patient care experience, especially given the uncertainties of COVID-19 persistence. Therefore, the current situation should encourage training coordinators in pharmacy schools to be innovative in creating training models that simulate direct patient care. Alternatives to real-world patient care could include the use of mock patient files from the system used in training facilities, virtual access to electronic medical records, interactive meetings with practicing pharmacists or APPE students, and attending telemedicine visits. These are all viable options that can be created, arranged, or simulated for students to experience patient care in virtual settings. However, there is an existing need to find the best match for virtual training styles that are appropriate for attaining specific patient care training objectives.

Although COVID-19 has had an impact on KSU’s pharmacy education and training, IPPE virtual training has had more than acceptable results, enabling students to complete their required hours of training without affecting their academic plan. However, the research and the virtual training program had some limitations that should be addressed in future research and training. While in-person interview would have been the best tool for data collection, especially for APPE students’ experience, the use of online qualitative questionnaire was the best alternative considering the novelty of this situation and the restrictions in place to limit the transition of COVID-19 infections. The study was conducted in one pharmacy school under unique conditions, which could limit the generalizability of the findings. Nevertheless, the study was conducted to provide a full overview of the virtual training program and did not assume that the findings would be the same in other schools. The program designed and administered in the absence of physical meetings between preceptors and/or APPE students. In addition, no extra resources were utilized, and the program was developed in a very short period of time due to the pandemic. Given the availability of more time and resources, developing extra patient care activities would improve the IPPE students’ training experience. The most significant drawbacks for this virtual IPPE training were the lack of actual direct patient care experience and the limited improvement on the IPPE student’s verbal communication skills. However, most of these IPPE students were Pharm. D students who would have enough time to fill this gap in experience and skills during their internship training.

## Conclusions

COVID-19 has impacted education and training worldwide and IPPE at KSU was no exception. The virtual training experience that has been developed and administered was not perfect, but it presented some advantages, challenges, and opportunities. Therefore, future training programs can be arranged to overcome the challenges and benefit from the opportunities. Moreover, future IPPE training does not need to be limited to traditional training but can be a mix of virtual and traditional methods to overcome the limited availability of training seats in hospitals and to limit the number of trainees per preceptor. The incorporation of telemedicine and virtual training could be used to meet the momentous need for training and to maximize the experiential benefits in the field of pharmacy training. Even in post-pandemic times, future training programs should incorporate online resources and simulation to facilitate and supplement students’ learning experience. Lastly, APPE students proved to be invaluable resources, and pharmacy schools should utilize their APPE students more often in administering courses and/or training for their fellow students. This both improves students’ learning outcomes and provides APPE students with the experience of supervising and training other pharmacy students after graduation.

## Supplementary Information

**Additional file 1 Table 1A**. The objectives for the actual and virtual institutional-IPPE training. **Table 2A**. Examples from APPE students’ responses to different aspects of the questionnaire. **Fig. 1A.** Flow diagram describing the steps and the APPE students’ roles in the preparation and implementation of the virtual IPPE training program.

## Data Availability

The datasets used and/or analyzed during the current study are not publicly available due the presence of student specific information but are available from the corresponding author on reasonable request.
