# Supplementary material for: Student and educator perspectives on virtual institutional introductory pharmacy practice experience (IPPE)
Source: BMC Med Educ. 2021 May 4;21:257. doi: 10.1186/s12909-021-02698-5 (PMC8093586; doi:10.1186/s12909-021-02698-5)
Supplement: Supplementary file 1 — Additional file 1 Table 1A. The objectives for the actual and virtual institutional-IPPE training. Table 2A. Examples from APPE students’ responses to different aspects of the questionnaire. Fig. 1A. Flow diagram describing the steps and the APPE students’ roles in the preparation and implementation of the virtual IPPE training program. [file 12909_2021_2698_MOESM1_ESM.pdf]

Supplementary Materials

Student and educator perspectives on virtual institutional introductory pharmacy practice experience (IPPE)

Omar A. Almohammed[a]

Lama H. Alotaibi[a]

Shatha A. Ibn Malik[a]

[a] Department of Clinical Pharmacy, College of Pharmacy, King Saud University, Riyadh, Saudi Arabia.

Table 1A. The objectives for the actual and virtual institutional-IPPE training

Actual Training	Online Training
• Understand the process of medications distribution, dispensing, compounding and purchasing in the setting of institutional pharmacy.<br>• Practice basic pharmaceutical care skills (patient medication history, counseling, drug information).<br>• Perform calculations required in pharmacy practice with accuracy and in a timely manner.<br>• Demonstrate knowledge of the intravenous admixture system and sterile preparations.<br>• Address issues related to medication safety at the site.<br>• Demonstrate knowledge of current standards of institutional pharmacy practice and operation.<br>• Demonstrate familiarity with brand and generic drug names, appearance, manufacturer, dosage form(s), and route of administration and therapeutic class for commonly-used drugs at the institution.<br>• Exhibit good communication skills during interactions with preceptors, patients, coworkers, and other health care professionals at the site.<br>• Display a positive attitude about the practice of pharmacy and the ability to problem-solve, and responsibility towards improving self- learning.<br>• Demonstrate a high standard of professional behavior (i.e., adhering to established work schedule, minimizing absences, respecting fellow colleagues).	♦ **Outpatient pharmacy**<br>• Understand different types of healthcare organizations and layout of outpatient pharmacy.<br>• Understand the process of medications distribution, dispensing in the setting of outpatient pharmacy.<br>• Demonstrate familiarity with pharmaceutical care practices in the outpatient setting (including patients' counseling, drug interactions, medication history, medication reconciliation).<br>• Demonstrate knowledge about e-prescribing as compared to hand-written prescription.<br>• Learn about patients' safety and medication errors from the outpatients' pharmacy setting perspective.<br><br>♦ **Inpatient pharmacy**<br>• Understand the process of medications distribution and dispensing in the setting of inpatient pharmacy.<br>• Demonstrate knowledge of the purpose, benefits, and challenges of using unit dose system.<br>• Demonstrate knowledge of the intravenous admixture system and sterile preparations.<br>• Demonstrate familiarity with compounding of pharmaceutical preparations in hospital setting.<br>• Practice basic skills related to drug information question and responding to these questions.<br>• Demonstrate knowledge of the process of hospitalized patients discharge and the role of pharmacists.<br>• Demonstrate knowledge of the emergency response team and the rule of pharmacists in these teams.<br>• Learn about patients' safety and medication errors from inpatients' pharmacy setting perspective.<br><br>♦ **Pharmacy management and soft skills**<br>• Understand the process of supply chain management for medications in hospitals.<br>• Demonstrate knowledge on how to store medications in different hospital pharmacy settings.<br>• Demonstrate knowledge of the role of P&T committee in hospitals and the need for medications formulary.<br>• Demonstrate familiarity with ethical and respectable practices in working environment.<br>• Learn about proper time management and teamwork.<br>• Complete a special COVID-19 training for health care workers and learn about proper infection control.

Table 2A. Examples from APPE students' responses to different aspects of the questionnaire

Overall experience

My experience was very good, but we found some difficulties in finding online courses that were freely accessible at that time. On the other hand, planning the activities and quizzes was fun to do.

I did enjoy reading and learning about the different aspects of the training objectives but, for me, preparing the course plan and material was extremely challenging due to the tight schedule.

It was a nice experience to participate in this course. We tried to make compatible materials that covered all the objectives in such a way that students would understand it.

Challenges and difficulties.

The time constraint and lack of overall insight into the course. We were thrown into the program at an awkward time in regards to the conception and realization of the course.

Access to some online resources through the school has expired which made it harder to obtain high-quality material in a short period of time.

The impact of previous experience on decisions when designing or selecting activities for the training

I used my previous knowledge and experience to create various activities. Before I chose the activities, I asked myself which kind of activities I enjoyed more during my studies so that my fellow students would enjoy them too.

The pharmacy practice lab was very helpful for me in this course.

We tried to prepare a similar experience to what we had. I have done many online courses before and this helped me to prepare the plan and material, I also had training for four months in a hospital.

Opportunities that APPE students had because of their experience in the virtual training

It gave me an opportunity to have an inside look into the preceptors' side of managing and grading students through the school learning management system.

I had the opportunity to choose the information that was relevant and important and, therefore, helped benefit the students.

I learned how to plan, prepare, conduct, administer, and assess the online training course for my fellow students.

Experiences of presenting the materials, constructing online assessments, and coordinating the virtual training

It was very entertaining and being involved in training students was a new and enriching experience.

Educational videos about how to deal with the school learning management system have helped us a lot. Assessing and evaluating student performance were interesting, I enjoyed doing that and writing notes for the students that may help them to improve their performance, know their mistakes, and try to fix them.

It was a new experience that I wanted to experience for a long time. It was fun, enjoyable, and I learned new things that I couldn't imagine learning.

Skills that APPE students gained or used during their experience in the virtual training

I believe my time management and efficient work skills helped me the most.

This rotation helped in refining my teaching skills and expand my interest in the academic field.

The most important skills are teamwork skills, especially communication skills.

Communication skills, patience, and problem-solving.

Pharmacy simulation program

I think it is a step in the right direction, it can simulate real-life scenarios.

The pharmacy simulation program was very helpful to teach dispensing skills to students.

Yes, of course I'd like to see more programs that are similar to this pharmacy simulation program, as these programs are considered more beneficial during online training.

Assessment of IPPE students' activities and providing feedback

I believe feedback is an essential part in learning. I didn't exactly learn from this as much as I got to demonstrate constructive criticism to my fellow students.

I learned how to grade the students and how to give professional feedback.

I would've liked if there was a grading rubric to ensure fairness when grading students.

Preferred learning style for the IPPE students from the APPE perspective

I believe the simulation, as it provided realistic scenarios and got the most positive feedback.

Lectures, they are the most important part which cover the main objectives.

I guess each student favored something, but mainly lectures and simulation where the most. I think it's because they are used to them more and the simulation because it's a new interesting style.

Preferred assessment style for the IPPE students from the APPE perspective

I believe that MCQ quizzes are best for measuring the student understanding of the material.

Simulation required the students to use and demonstrate what they learned in a way that shows their understanding.

Team-based activity, because the student will share the knowledge and skills with the team.

Would you join or recommend online training courses to others in the future?

Yes, for sure! It was my best experience in a long time.

Yes, because of the flexibility in time, as learning and teaching can take place in any location.

No, I think actual training is more beneficial than online training.

No, online training should always be voluntary and not provide any form of certification due to the lack of enforcement of standardization.

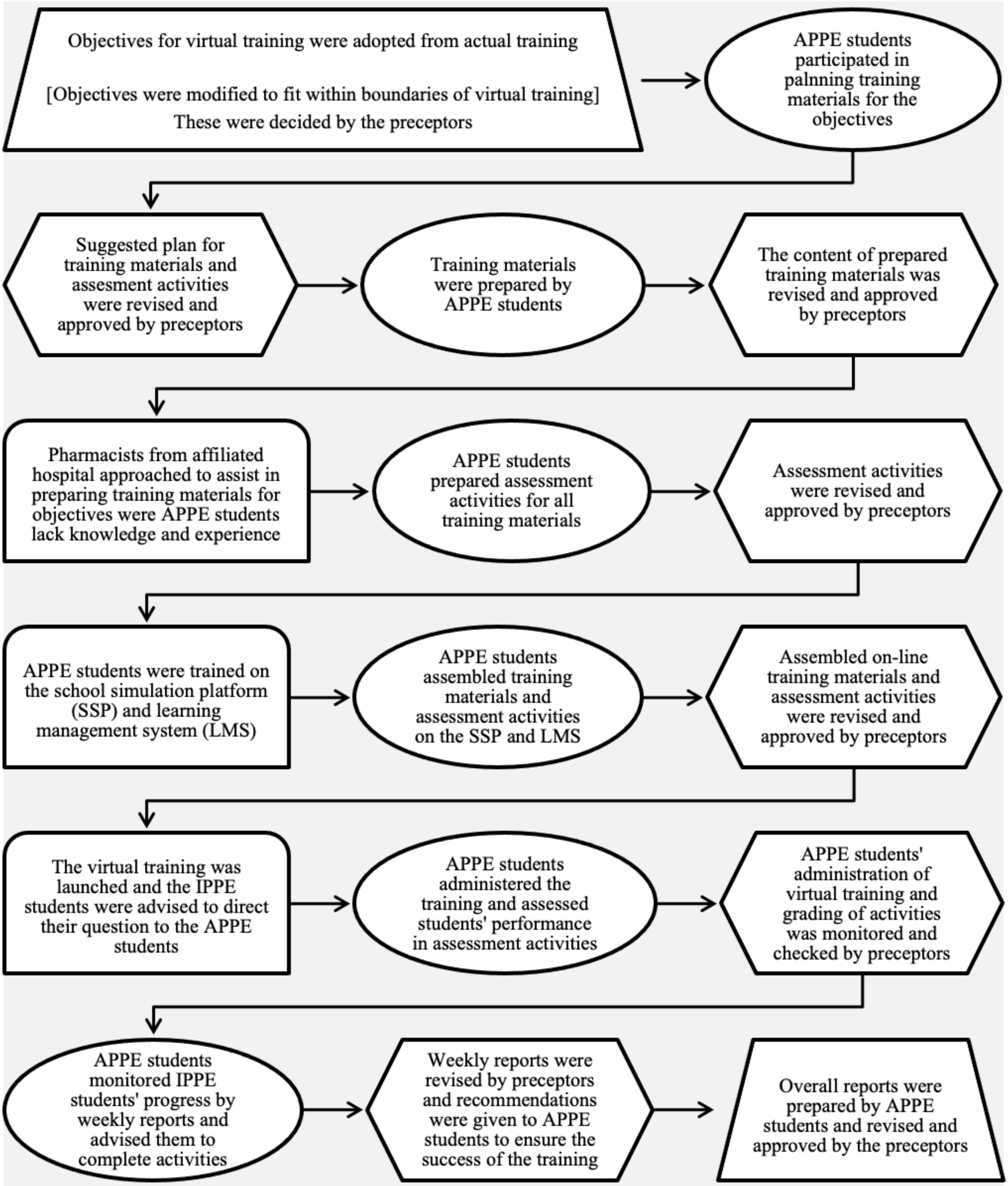

Figure 1A. Flow diagram describing the steps and the APPE students' roles in the preparation and implementation of the virtual IPPE training program
